# Supplementary material for: Synthetic Tryptanthrin Derivatives Induce Cell Cycle Arrest and Apoptosis via Akt and MAPKs in Human Hepatocellular Carcinoma Cells
Source: Biomedicines. 2021 Oct 24;9(11):1527. doi: 10.3390/biomedicines9111527 (PMC8615277; doi:10.3390/biomedicines9111527)
Supplement: Supplementary file 1 [file biomedicines-09-01527-s001.zip › biomedicines-1374207-supplementary.pdf]

Supplementary materials

Synthetic tryptanthrin derivatives induce cell cycle arrest and apoptosis via Akt and MAPKs in human hepatocellular carcinoma cells

Jing-Yan Gao[1], Chih-Shiang Chang[1,2], Jin-Cherng Lien[1,2], Ting-Wei Chen[2], Jing-Lan Hu[3] and Jing-Ru Weng[3,4,*]

[1]School of Pharmacy, China Medical University, Taichung 404020, Taiwan

[2]Drug Development Center, China Medical University, Taichung 404020, Taiwan

[3]Department of Marine Biotechnology and Resources, National Sun Yat-sen University, Kaohsiung 80424, Taiwan

[4]Graduate Institute of Natural Products, Kaohsiung Medical University, Kaohsiung 80708, Taiwan

Table of Contents

	Page
**General materials and methods for synthesis**	S3
**Procedures for synthesis of tryptanthrin and compound A1-A12**	S4-S9
**^{1}H NMR spectra of tryptanthrin and compound A1-A12**	S10-S29
**HPLC spectra of tryptanthrin and compound A1-A12**	S10-S29
**Mass spectra of tryptanthrin and compound A1-A12**	S11-S29

General materials and methods for synthesis

All chemical reagents and solvents were commercial available. The reactions were monitored by thin layer chromatography (TLC) which on aluminium backed plates coated with Merck 60 F_{254} silica gel. Flash column chromatography was done by using silica gel (Merck Kieselgel 60, No. 9385, 230-400 mesh). ^{1}H NMR spectra were recorded at 400 MHz NMR spectrometer, all the samples were dissolved in Dimethyl sulfoxide-d6. Mass spectra were measured using a MAT 95 XL Thermo Quest Finnigan (Thermo Fisher Scientific). Chromatographic conditions: The separation was performed on C_{18} (COSMOSIL 5C18 AR-II-C_{18} Column; 250 × 4.6 mm, 5 μm) using a mixture of water/acetonitrile solution as a mobile phase with gradient elution mode. The injection column was 10 μl with UV scanning at 252 nm at column oven 25℃. The chromatographic run time was 35 min.

Procedures for synthesis of tryptanthrin and compound A1-A12

Corresponding isatin derivatives (1 mmol.) and isatoic anhydride (1 mmol.) were combined with toluene (25 mL) in a 150 ml round bottomed flask equipped with a stir bar. The triethylamine (5 mmol) was added into the reaction mixture and reflux for 6 h (Scheme S1). After cooling, the reaction mixture was concentrated in vacuo to remove the toluene. The resulting precipitates were taken into ethyl acetate (250 mL) and

washed with brine (2 × 100 mL). The organic phase was dried by anhydrous Magnesium Sulfate and evaporated under reduced pressure. The residue was purified by flash column chromatography to give **A1**-**A12**.

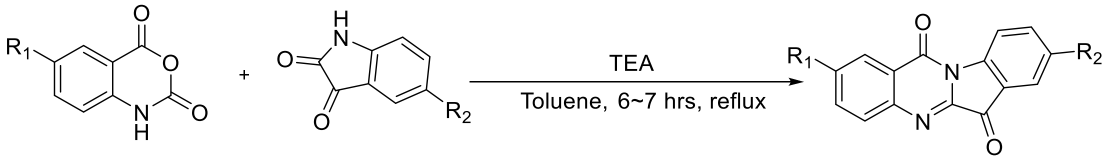

Scheme S1. General procedure for synthesis of tryptanthrin and compound **A1-A12**.

Indolo[2,1-*b*]quinazoline-6,12-dione (Tryptanthrin).

Yellow crystal; 76 % yield. ^{1}H NMR (400 MHz, DMSO) δ 8.46 (d, *J* = 7.9 Hz, 2H), 8.30 (d, *J* = 7.8 Hz, 1H), 7.93 (d, *J* = 3.5 Hz, 2H), 7.89 – 7.81 (m, 4H), 7.77 – 7.68 (m, 2H), 7.47 (t, *J* = 7.5 Hz, 1H). MS (ESI) calculated for Chemical Formula: $C_{15}H_8N_2O_2$; Molecular Weight: 248.24; Found 248.1.

8-bromoindolo[2,1-*b*]quinazoline-6,12-dione (A1)

Ochre crystal; 70% yield; ^{1}H NMR (400 MHz, DMSO) δ 8.44 – 8.37 (m, 1H), 8.32 (dt, *J* = 7.8 Hz, 1H), 8.08 – 8.01 (m, 2H), 7.98 – 7.91 (m, 2H), 7.74 (dt, *J* = 8.2, 4.2 Hz,

1H). MS (ESI) calculated for Chemical Formula: $C_{15}H_7BrN_2O_2$; Molecular Weight: 327.14; Found 326.0.

8-iodoindolo[2,1-*b*]quinazoline-6,12-dione (A2)

Ochre crystal; 72% yield; ^{1}H NMR (400 MHz, DMSO) δ 8.33 – 8.21 (m, 1H), 8.21 – 8.13 (m, 1H), 7.99 – 7.90 (m, 1H), 7.78 – 7.67 (m, 1H). MS (ESI) calculated for Chemical Formula: $C_{15}H_7IN_2O_2$; Molecular Weight: 374.14; Found 374.0.

8-chloroindolo[2,1-*b*]quinazoline-6,12-dione (A3)

Ochre crystal; 68% yield; ^{1}H NMR (400 MHz, DMSO) δ 8.46 (d, *J* = 8.5 Hz, 1H), 8.32 (d, *J* = 7.9 Hz, 1H), 7.98 – 7.93 (m, 3H), 7.91 (d, *J* = 8.5 Hz, 1H), 7.74 (dt, *J* = 8.1 Hz, 1H). MS (ESI) calculated for Chemical Formula: $C_{15}H_7ClN_2O_2$; Molecular Weight: 282.68; Found 282.1.

8-fluoroindolo[2,1-*b*]quinazoline-6,12-dione (A4)

Yellow crystal; 65% yield; ^{1}H NMR (400 MHz, DMSO) δ 8.48 (dd, *J* = 8.8, 4.2 Hz, 1H), 8.31 (dt, *J* = 7.8, 1.1 Hz, 1H), 7.99 – 7.89 (m, 2H), 7.81 – 7.67 (m, 3H). MS (ESI) calculated for Chemical Formula: $C_{15}H_7FN_2O_2$; Molecular Weight: 266.23; Found

266.1.

8-methylindolo[2,1-*b*]quinazoline-6,12-dione (A5)

Yellow crystal; 72% yield; ^{1}H NMR (400 MHz, DMSO) δ 8.29 (dd, *J* = 11.5, 8.2 Hz, 2H), 7.92 (d, *J* = 4.1 Hz, 2H), 7.71 (dt, *J* = 8.2, 4.2 Hz, 1H), 7.65 (d, *J* = 12.7 Hz, 2H), 2.39 (s, 3H). MS (ESI) calculated for Chemical Formula: $C_{16}H_{10}N_2O_2$; Molecular Weight: 262.27; Found 262.1.

8-nitroindolo[2,1-*b*]quinazoline-6,12-dione (A6)

Brown crystal; 51% yield; ^{1}H NMR (400 MHz, DMSO) δ 8.75 – 8.63 (m, 2H), 8.54 (d, *J* = 2.3 Hz, 1H), 8.35 (d, *J* = 7.9 Hz, 1H), 8.04 – 7.94 (m, 2H), 7.81 – 7.73 (m, 1H). MS (ESI) calculated for Chemical Formula: $C_{15}H_7N_3O_4$; Molecular Weight: 293.24; Found 293.2.

Scheme S2. Synthesis of A7.

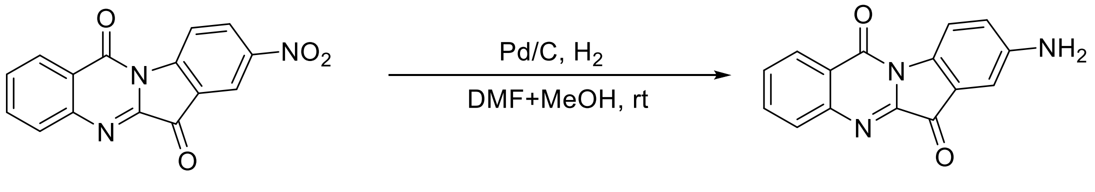

A mixture of **A6** (400 mg), 10% Palladium on Carbon and Hydrogen (atmospheric) in DMF (100 ml) and MeOH (50 ml) at room temperature for 4 h (Scheme S2), and the reaction was monitored by TLC. After completion, the reaction mixture was filtered by celite to obtain the final product (**A7**).

8-aminoindolo[2,1-*b*]quinazoline-6,12-dione (A7)

Deep purple crystal; 50% yield; ^{1}H NMR (400 MHz, DMSO) δ 8.25 (d, *J* = 7.6 Hz, 1H), 8.12 (d, *J* = 9.6 Hz, 1H), 7.96 – 7.79 (m, 2H), 7.74 – 7.64 (m, 1H), 7.01 – 6.93 (m, 2H), 5.64 (s, 2H); MS (ESI) calculated for Chemical Formula: $C_{15}H_9N_3O_2$. Molecular Weight: 263.26; Found 263.1.

8-(trifluoromethyl)indolo[2,1-*b*]quinazoline-6,12-dione (A8)

Brown crystal; 50% yield; ^{1}H NMR (400 MHz, DMSO) δ 8.66 (d, *J* = 8.4 Hz, 1H), 8.35 (dt, *J* = 7.9, 1.1 Hz, 1H), 8.29 – 8.19 (m, 2H), 8.03 – 7.93 (m, 2H), 7.82 – 7.71 (m, 1H). MS (ESI) calculated for Chemical Formula: $C_{16}H_7F_3N_2O_2$; Molecular Weight: 316.24; Found 316.1.

Scheme S3. Synthesis of A9.

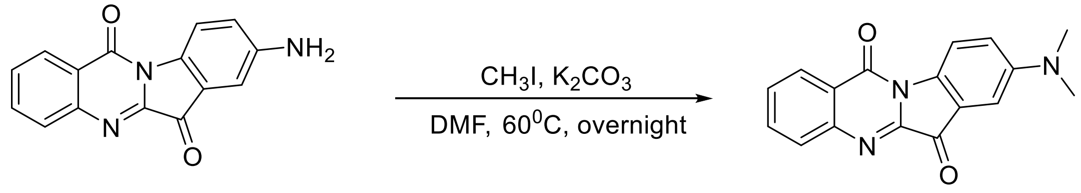

A mixture of (**A7**) (1 mmol), CH_3I (4.5 mmol), and K_2CO_3 (6.5 mmol) in DMF was heated on 60℃ overnight (Scheme S3), and the reaction was monitored by TLC. At completion, the solid residue was partitioned between ethyl acetate and water, and the resulting precipitates in both organic and aqueous phases were collected by filtration to obtain the final product (**A9**) (95% purity per ^{1}H NMR) without going through further purification.

8-(dimethylamino)indolo[2,1-*b*]quinazoline-6,12-dione (A9)

Purple crystal; 14% yield; ^{1}H NMR (400 MHz, DMSO) δ 8.29 – 8.14 (m, 2H), 7.91 – 7.86 (m, 2H), 7.69 (dt, *J* = 8.5, 4.2 Hz, 1H), 7.14 (d, *J* = 9.1 Hz, 1H), 7.04 (s, 1H), 2.97 (s, 6H). MS (ESI) calculated for Chemical Formula: $C_{17}H_{13}N_3O_2$; Molecular Weight: 291.31; Found 291.2.

2-bromoindolo[2,1-*b*]quinazoline-6,12-dione (A10)

Yellow crystal; 76% yield; ^{1}H NMR (400 MHz, DMSO) δ 8.46 (d, *J* = 8.0 Hz, 1H), 8.39 (s, 1H), 8.11 (d, *J* = 8.5 Hz, 1H), 7.92 – 7.83 (m, 3H), 7.49 (t, *J* = 7.5 Hz, 1H). MS

(ESI) calculated for Chemical Formula: $C_{15}H_7BrN_2O_2$; Molecular Weight: 327.14; Found 326.0.

2-fluoroindolo[2,1-*b*]quinazoline-6,12-dione (A11)

Yellow crystal;71% yield; ^{1}H NMR (400 MHz, DMSO) δ 8.47 (d, *J* = 7.9 Hz, 1H), 8.08 – 8.00 (m, 2H), 7.92 – 7.79 (m, 3H), 7.49 (t, *J* = 7.5 Hz, 1H). MS (ESI) calculated for Chemical Formula: $C_{15}H_7FN_2O_2$; Molecular Weight: 266.23; Found 266.1.

8-bromo-2-fluoroindolo[2,1-*b*]quinazoline-6,12-dione (A12)

Yellow crystal; 81% yield; ^{1}H NMR (400 MHz, DMSO) δ 8.39 (d, *J* = 8.4 Hz, 1H), 8.10 – 7.98 (m, 4H), 7.85 (td, *J* = 8.6, 3.0 Hz, 1H). MS (ESI) calculated for Chemical Formula: $C_{15}H_6BrFN_2O_2$; Molecular Weight: 345.13; Found 344.0.

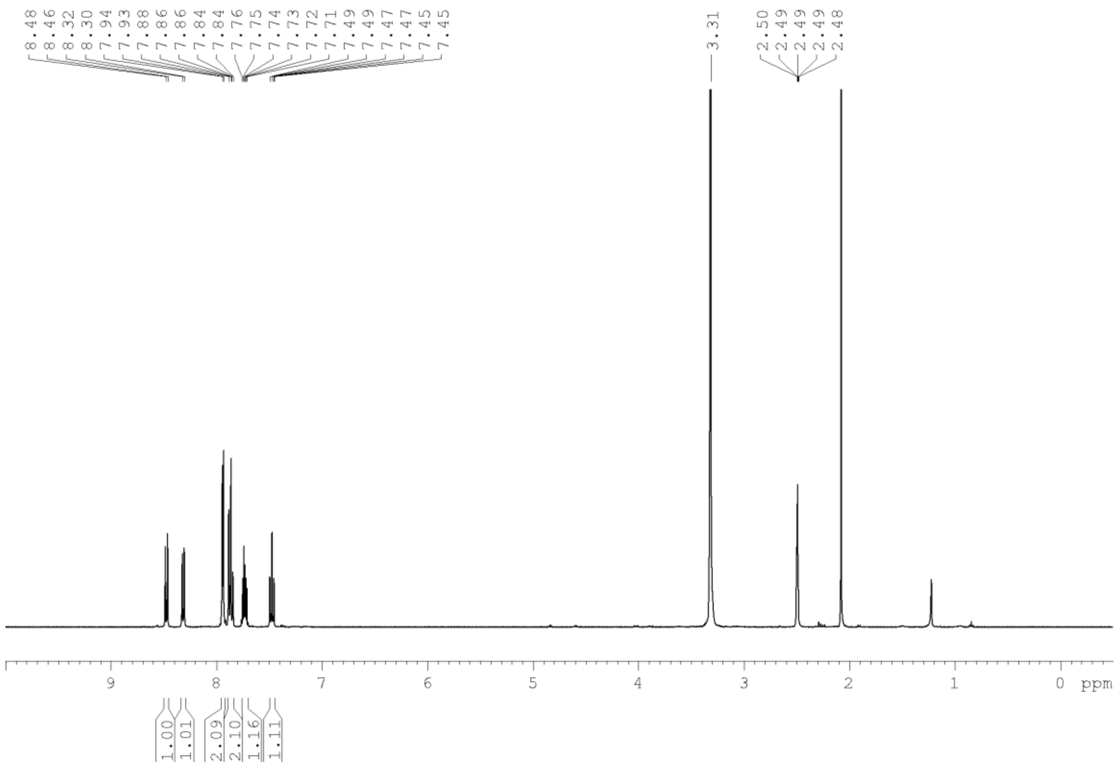

^{1}H NMR spectra of tryptanthrin

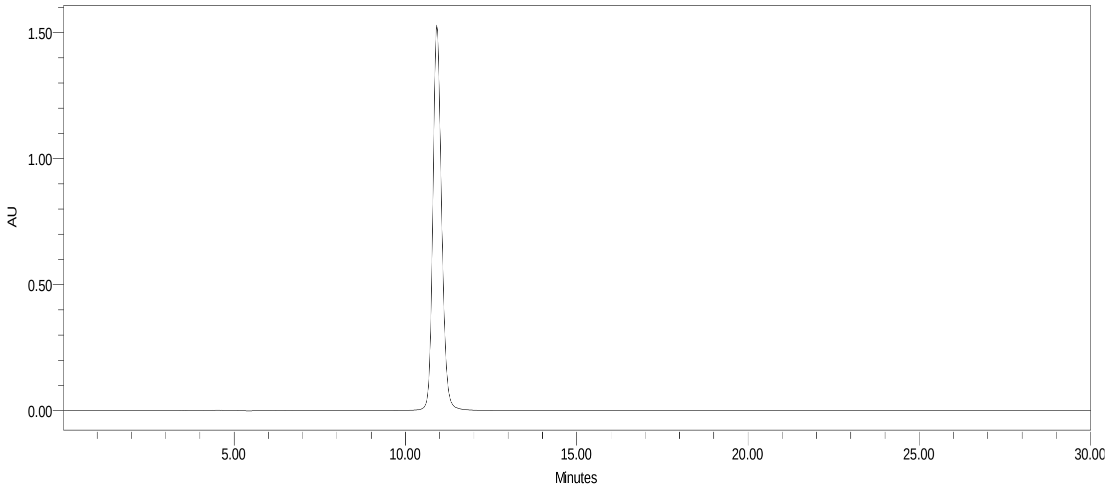

HPLC spectra of tryptanthrin

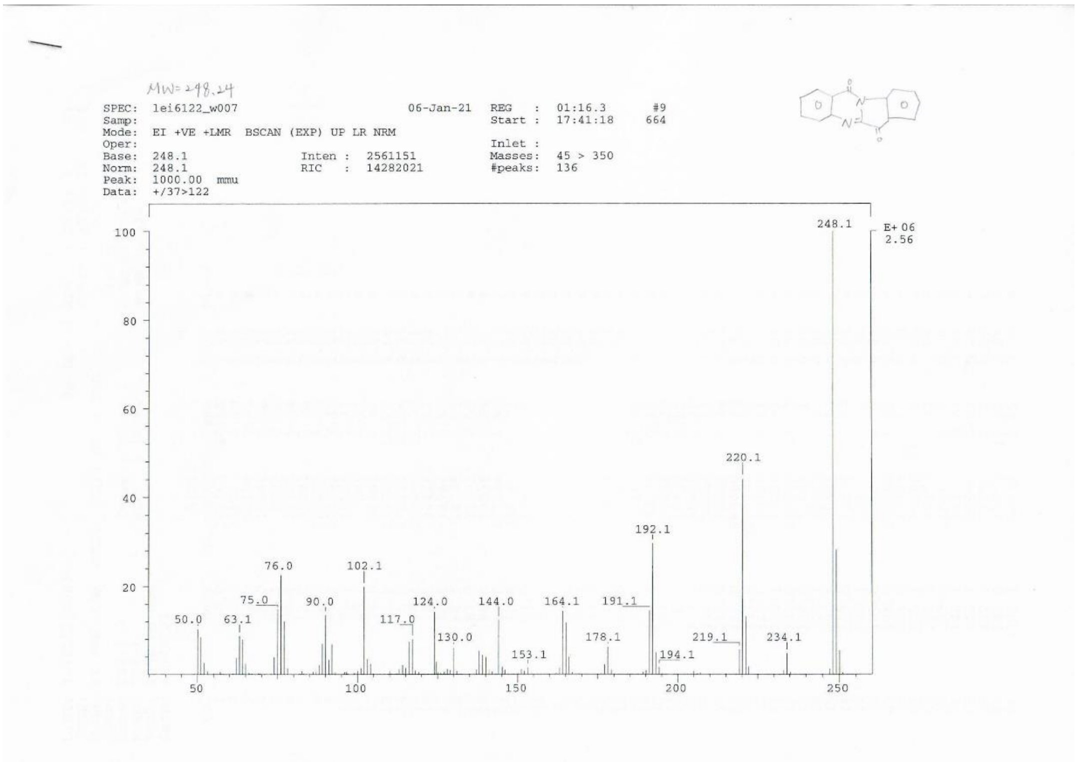

Mass spectra of tryptanthrin

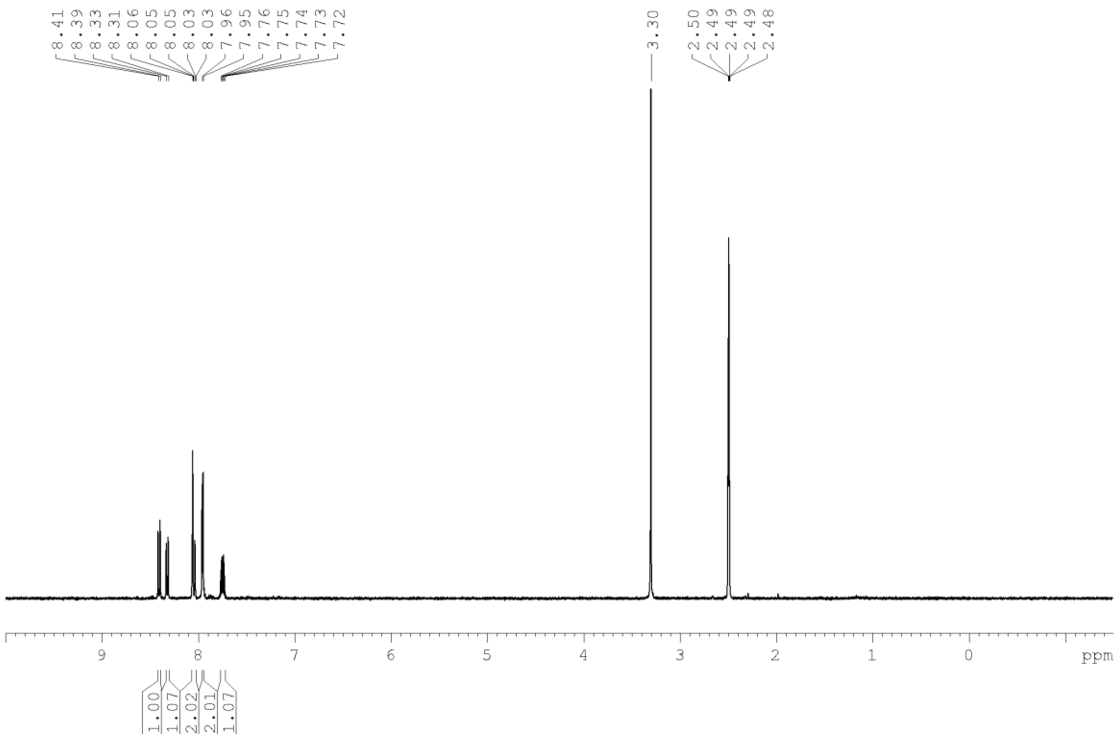

^{1}H NMR spectra of A1

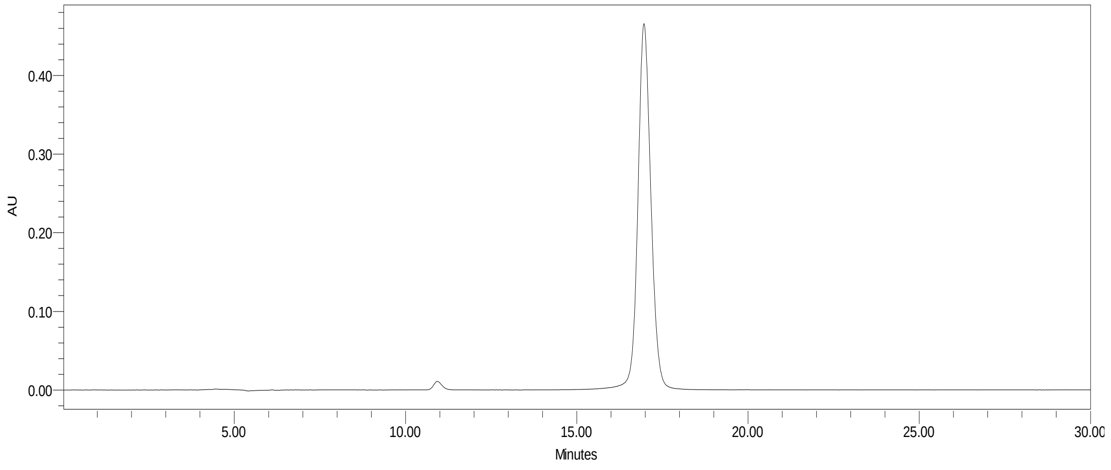

HPLC spectra of A1

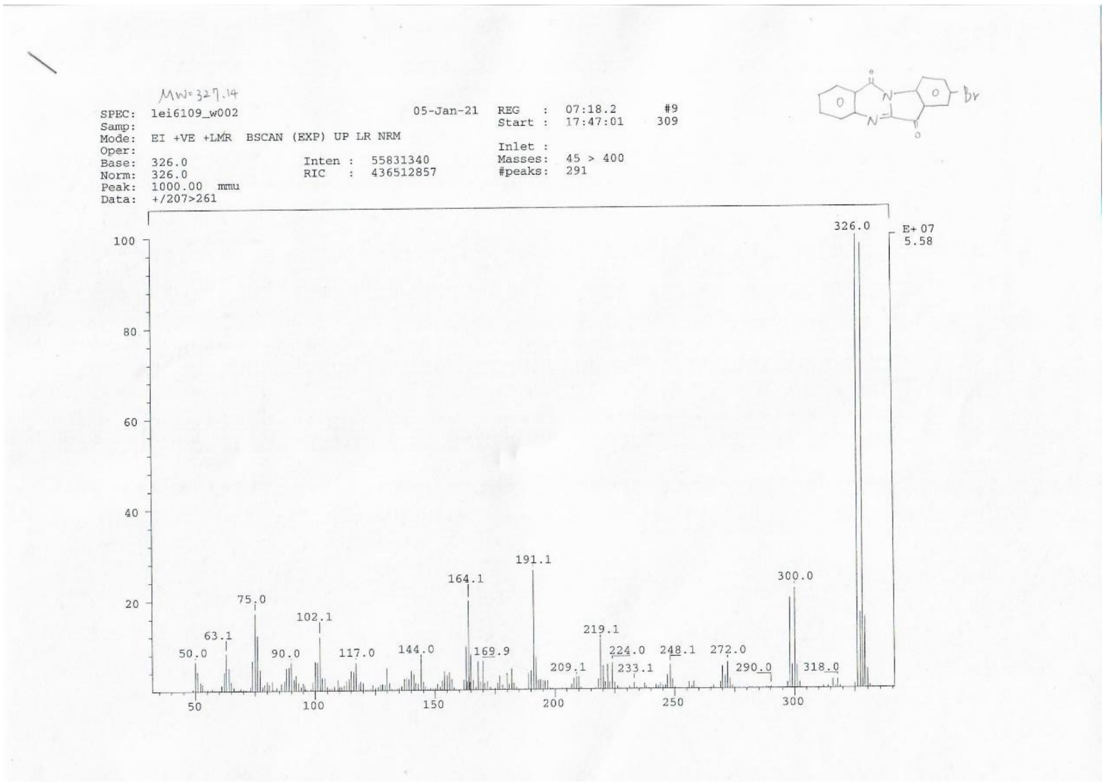

Mass spectra of A1

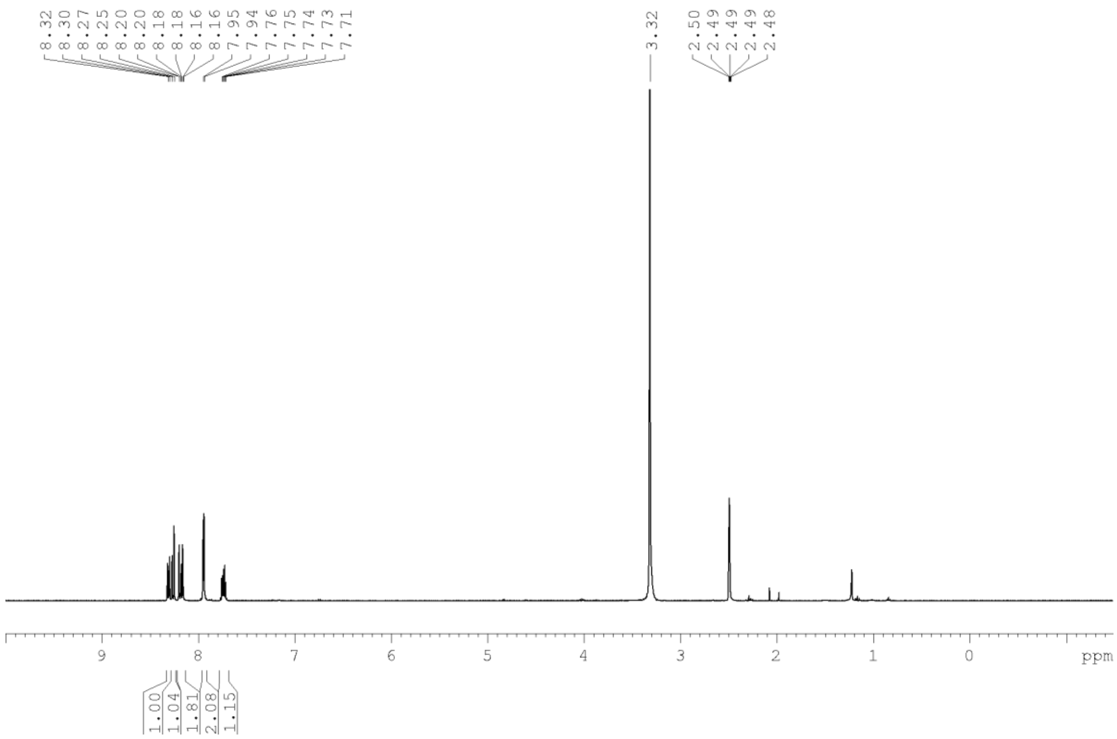

^{1}H NMR spectra of A2

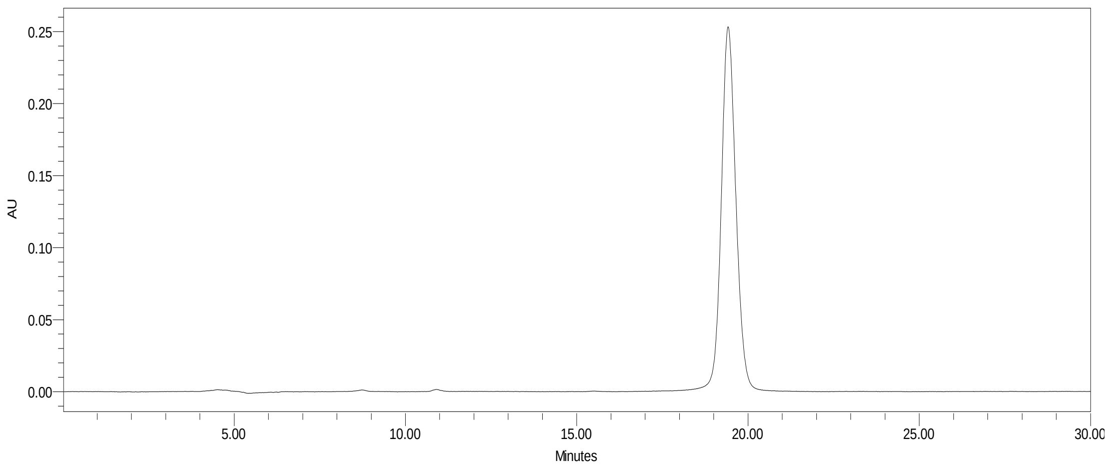

HPLC spectra of A2

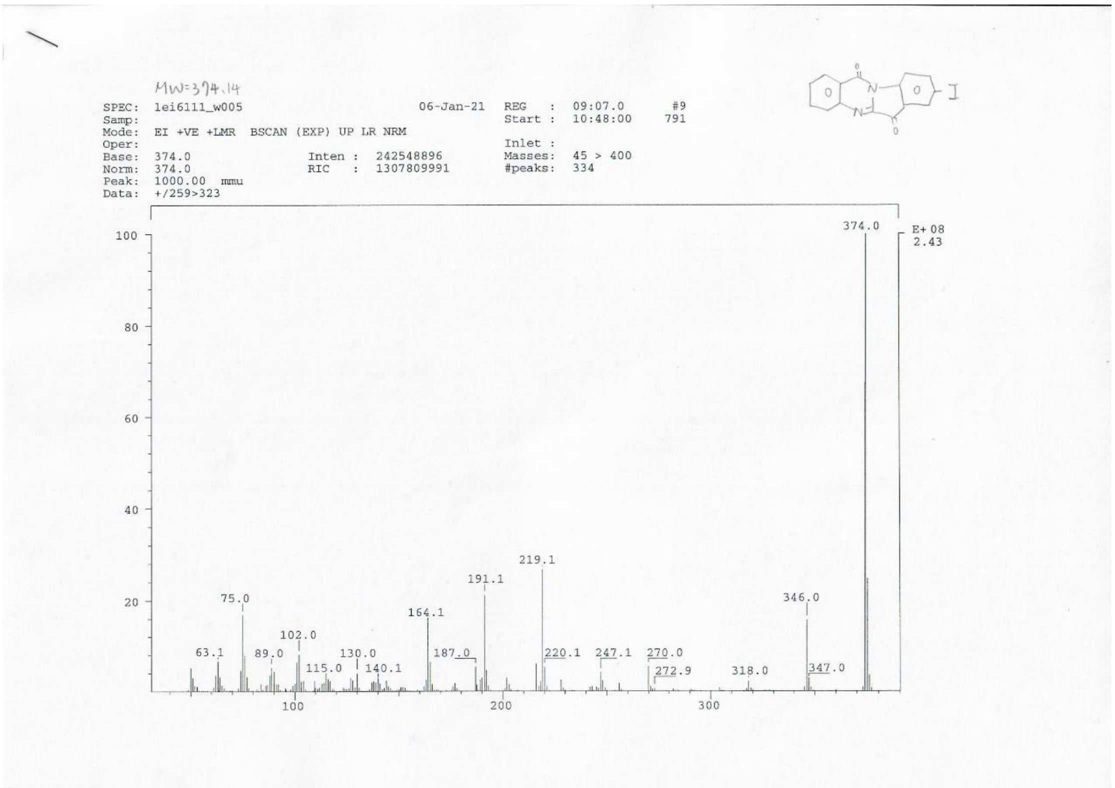

Mass spectra of A2

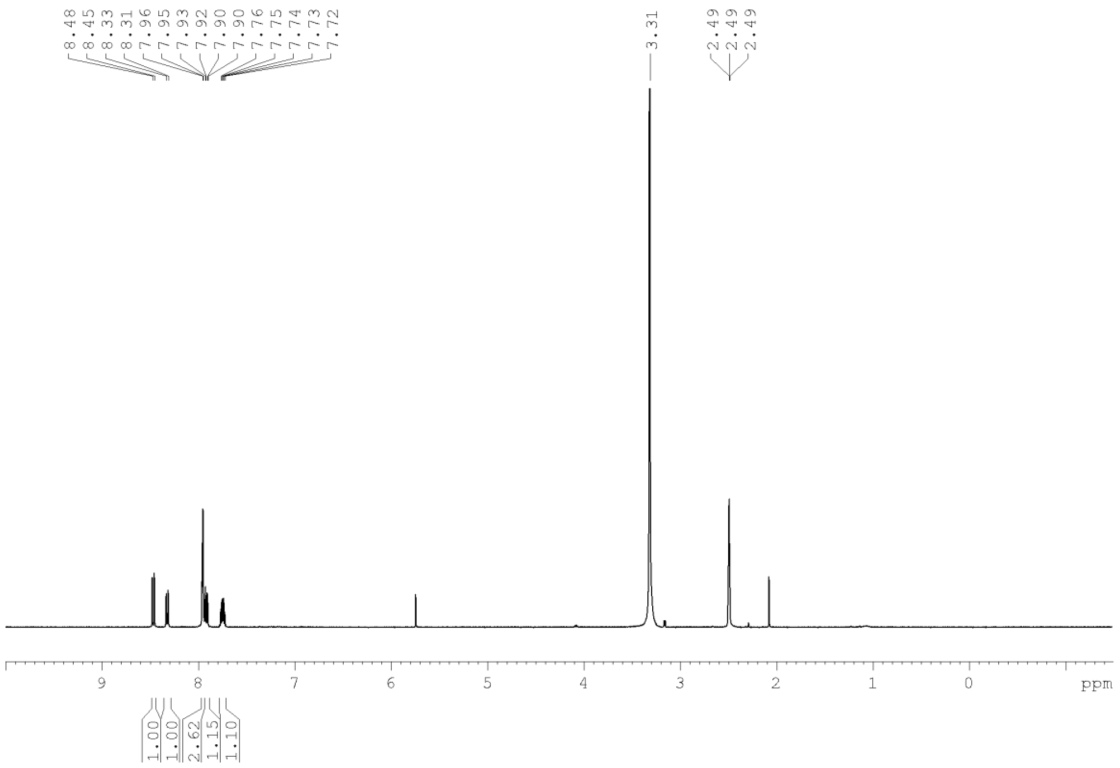

^{1}H NMR spectra of A3

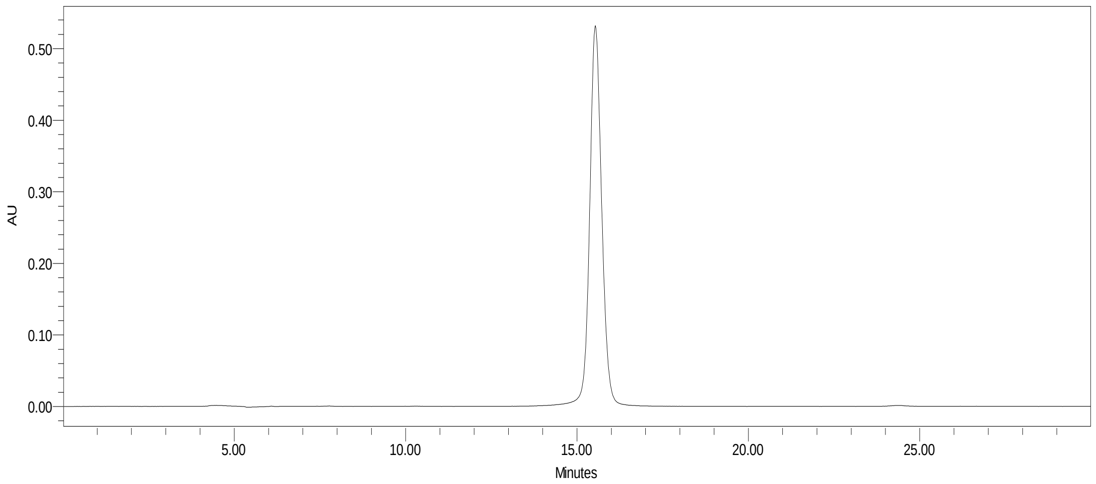

HPLC spectra of A3

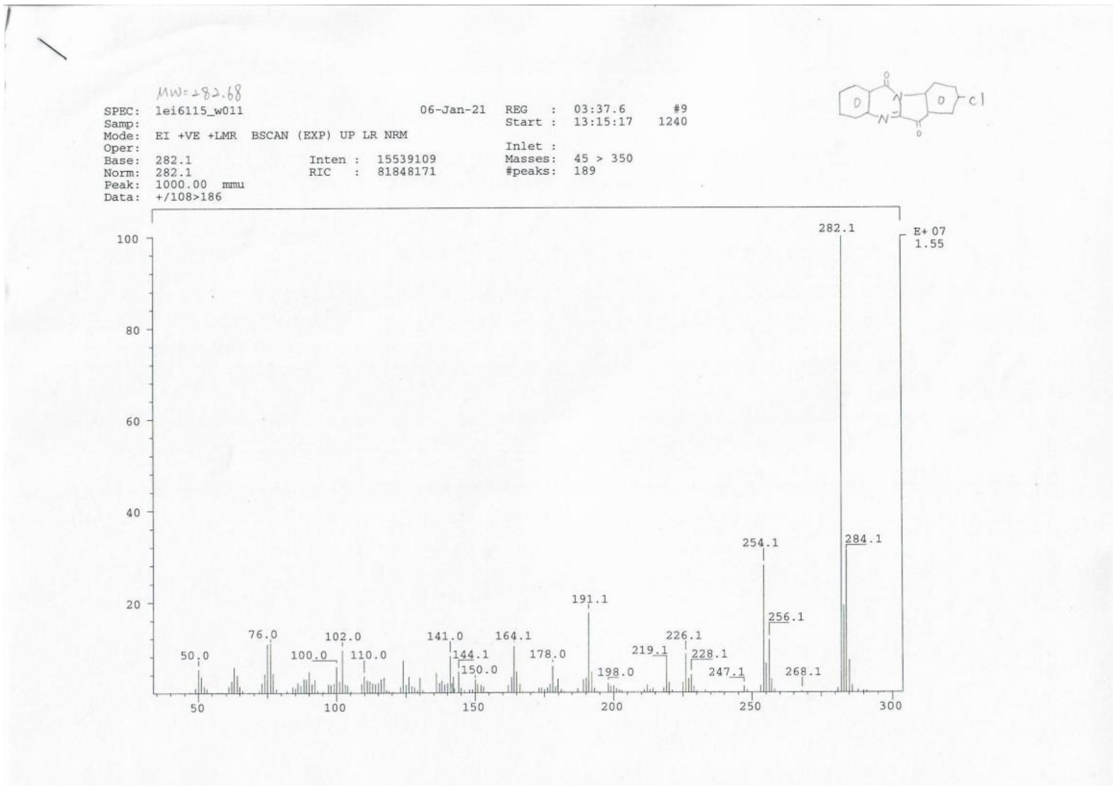

Mass spectra of A3

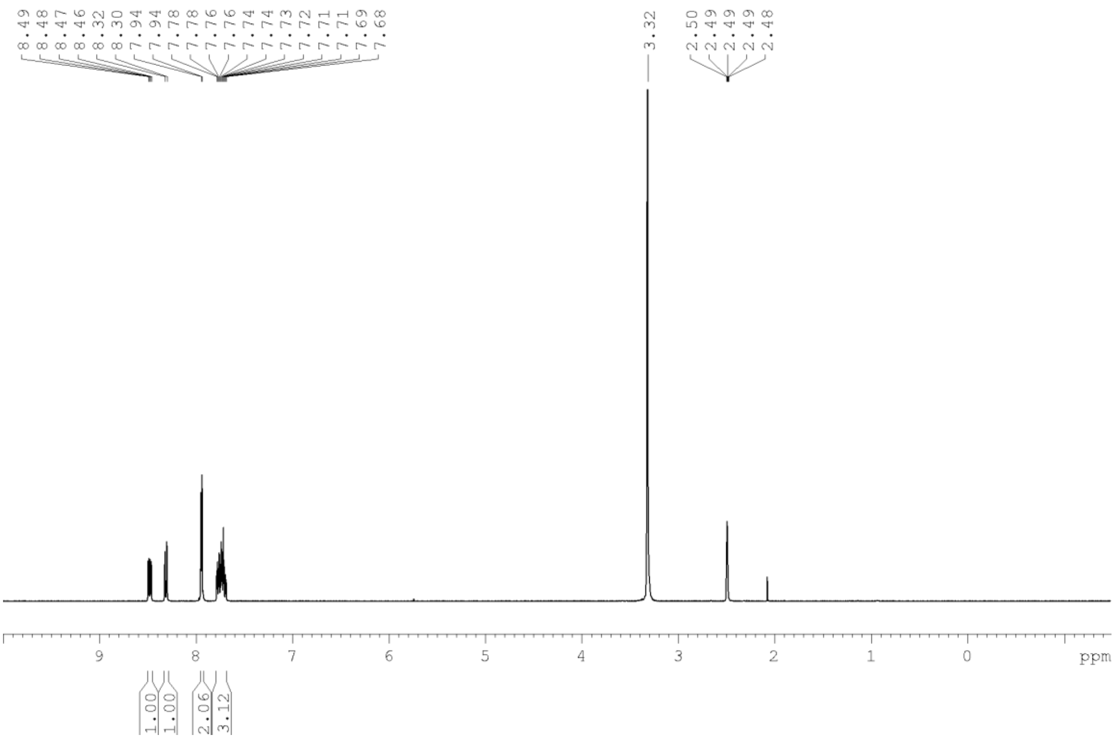

^{1}H NMR spectra of A4

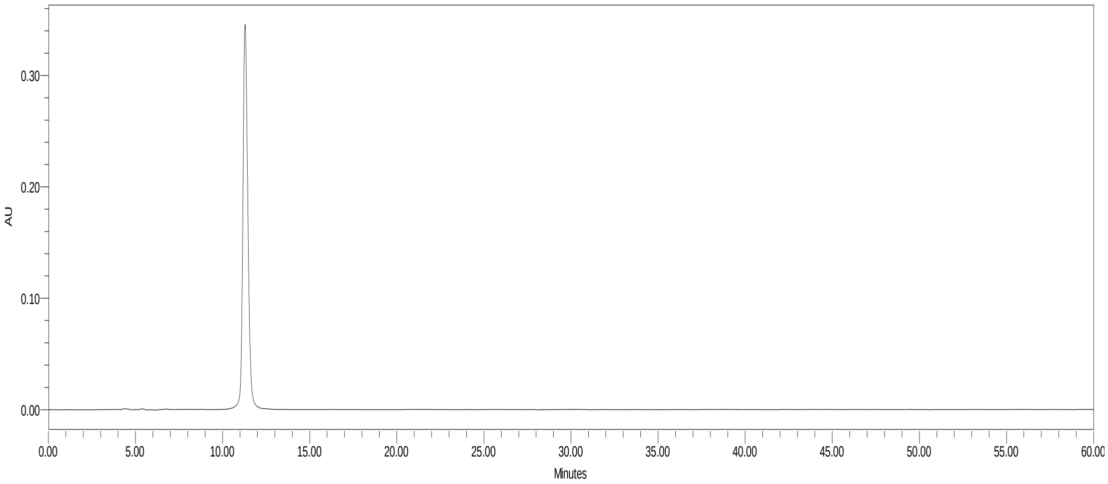

HPLC spectra of A4

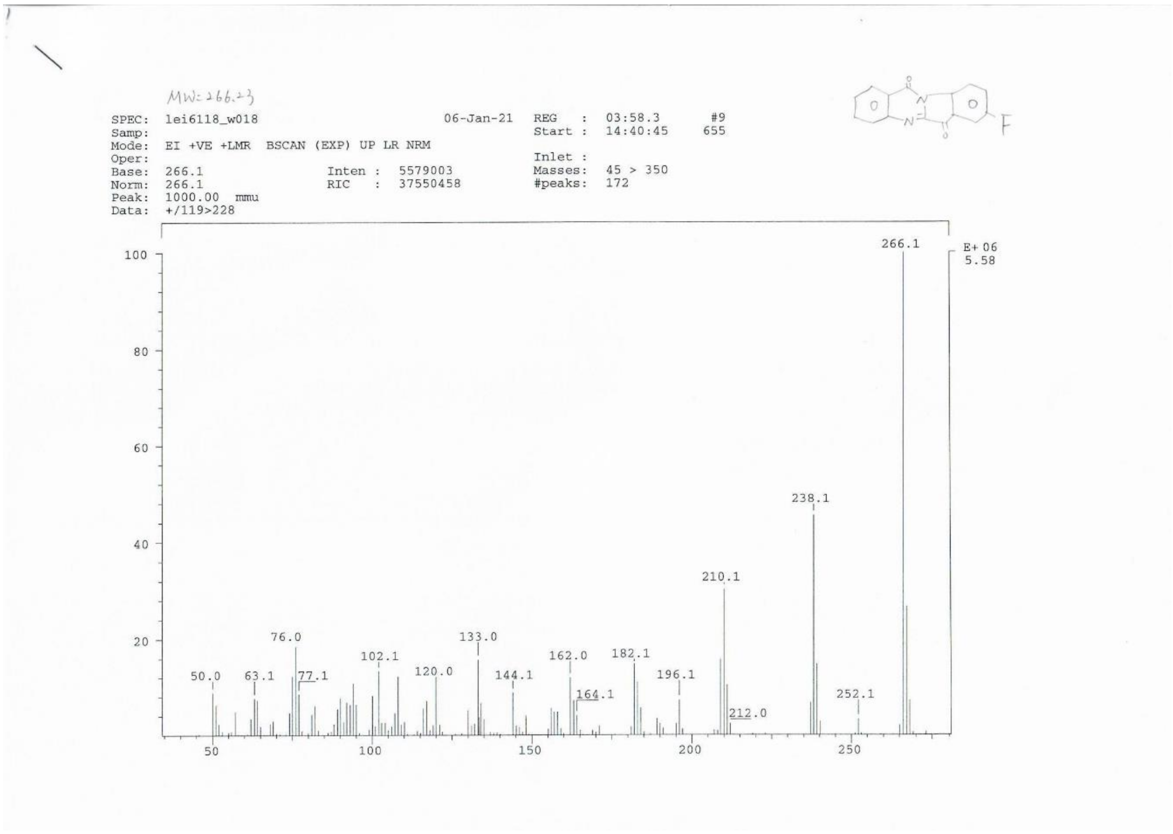

Mass spectra of A4

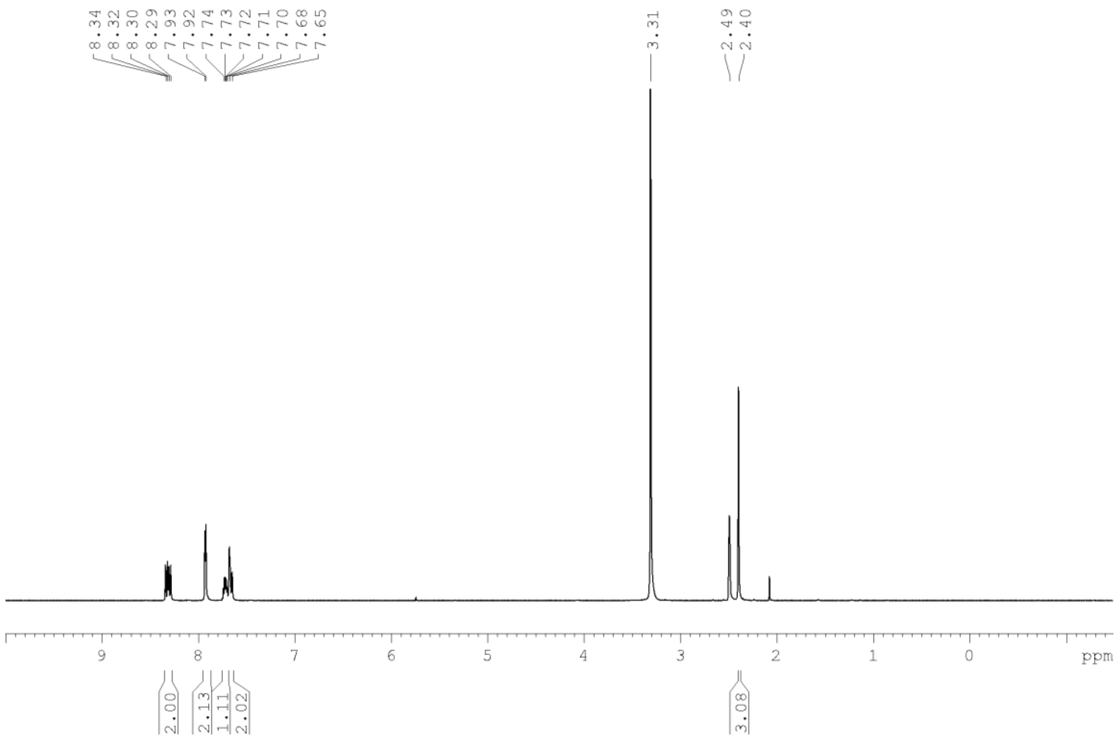

^{1}H NMR spectra of A5

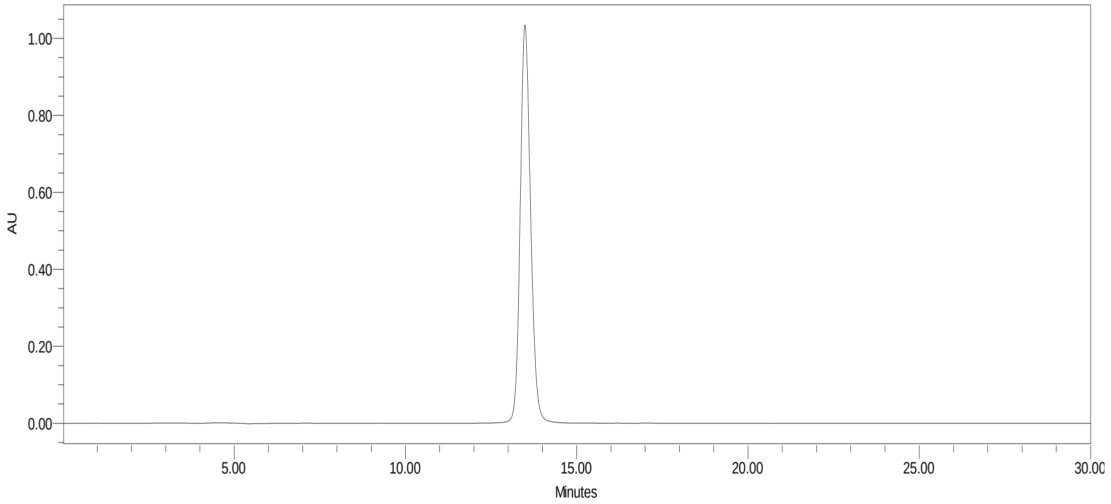

HPLC spectra of A5

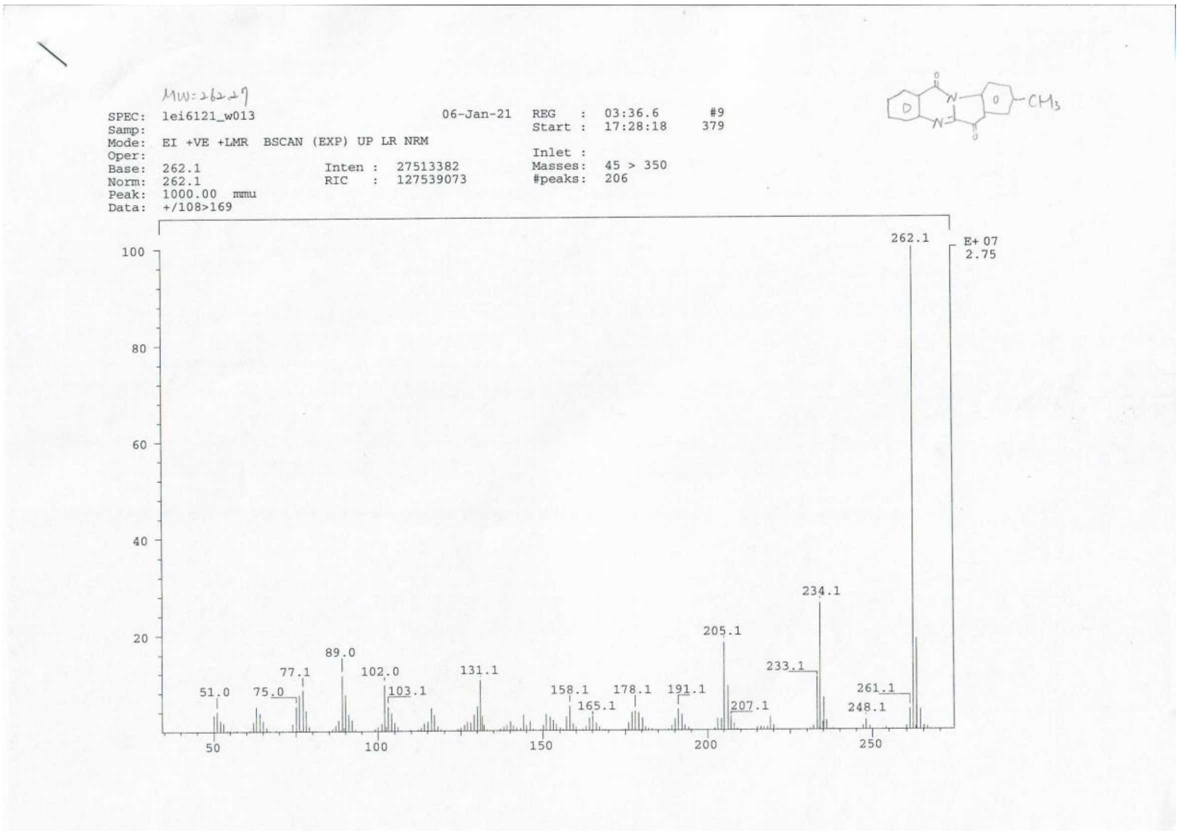

Mass spectra of A5

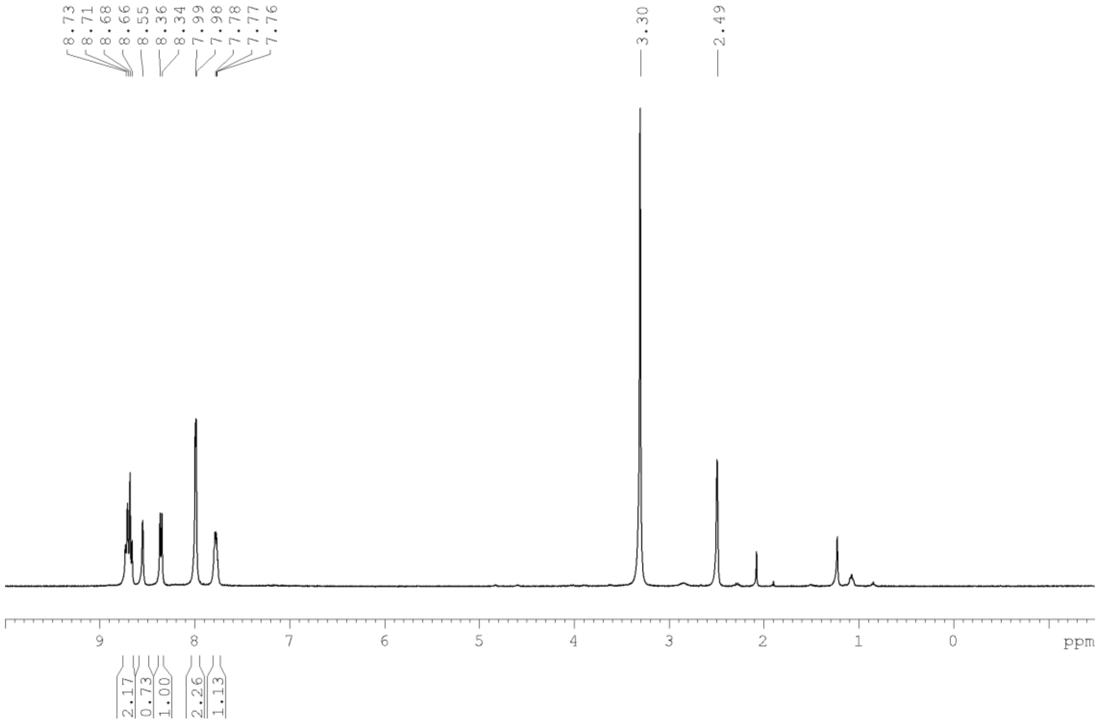

^{1}H NMR spectra of A6

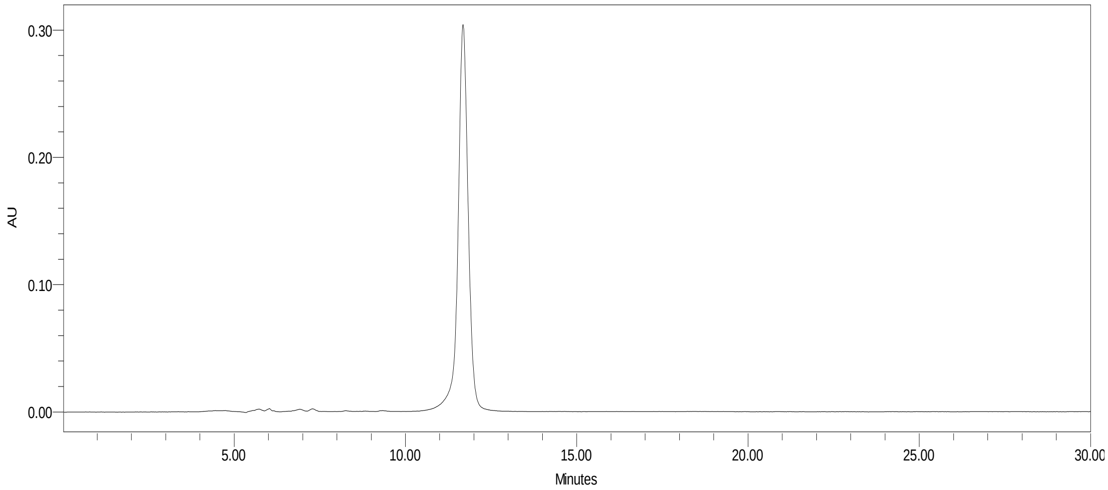

HPLC spectra of A6

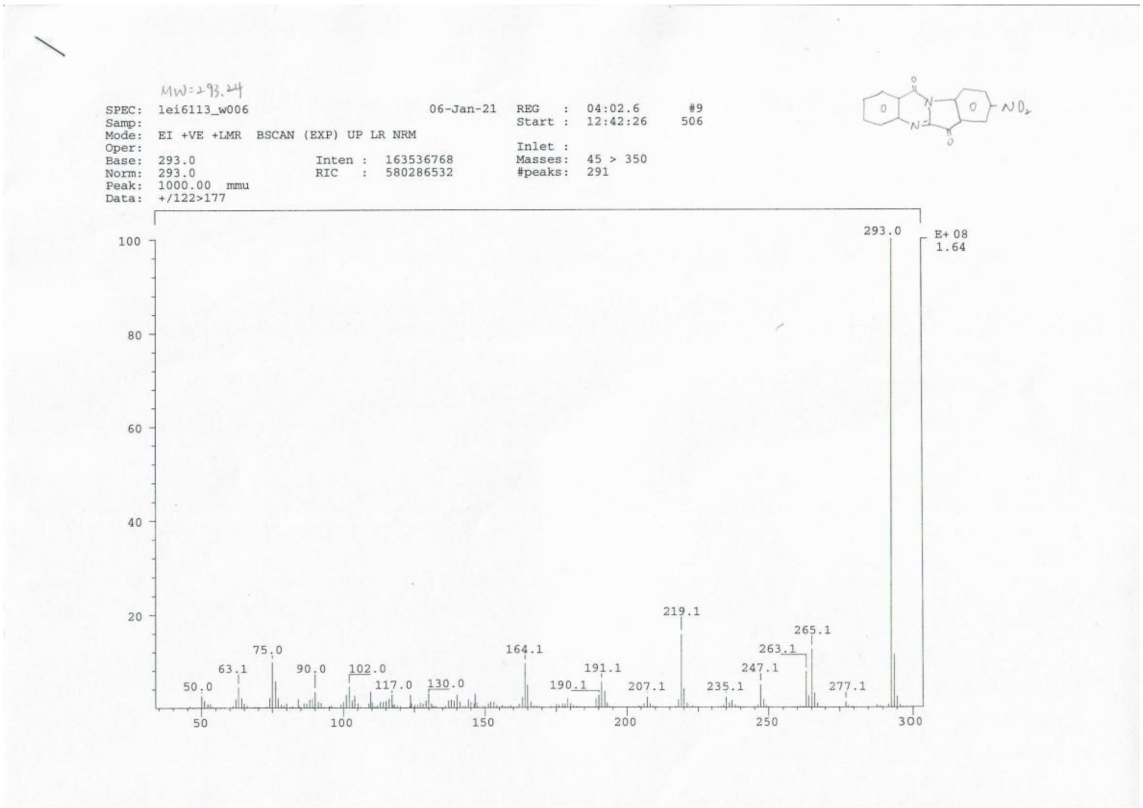

Mass spectra of A6

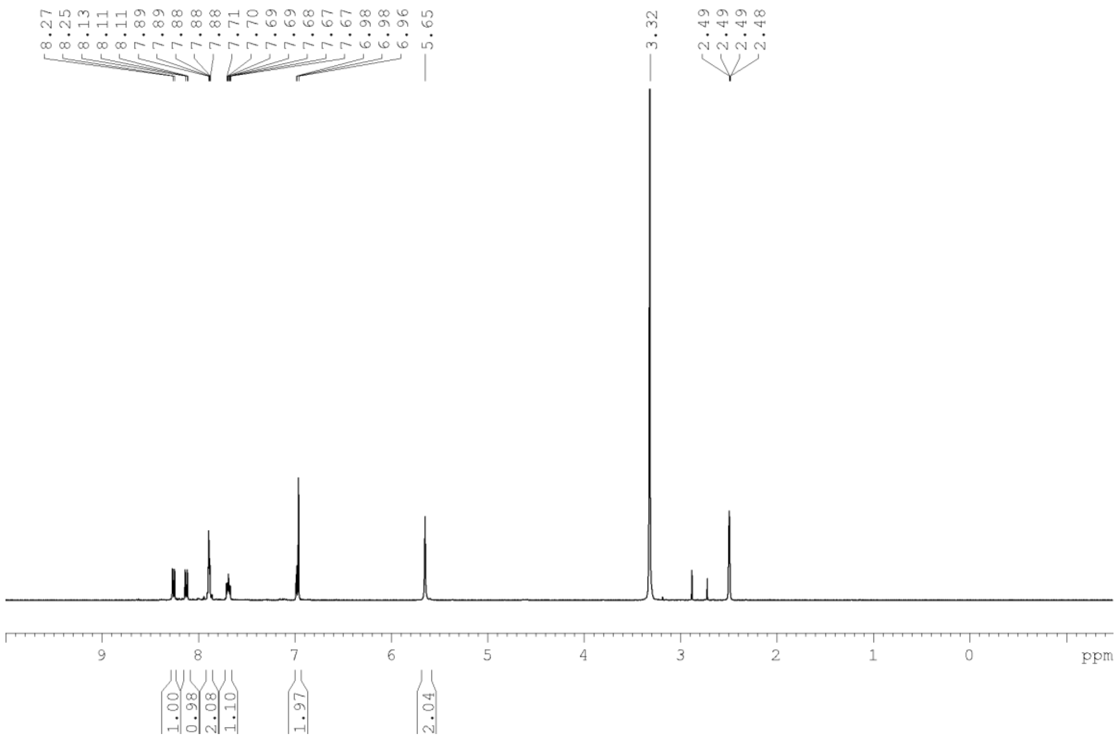

^{1}H NMR spectra of A7

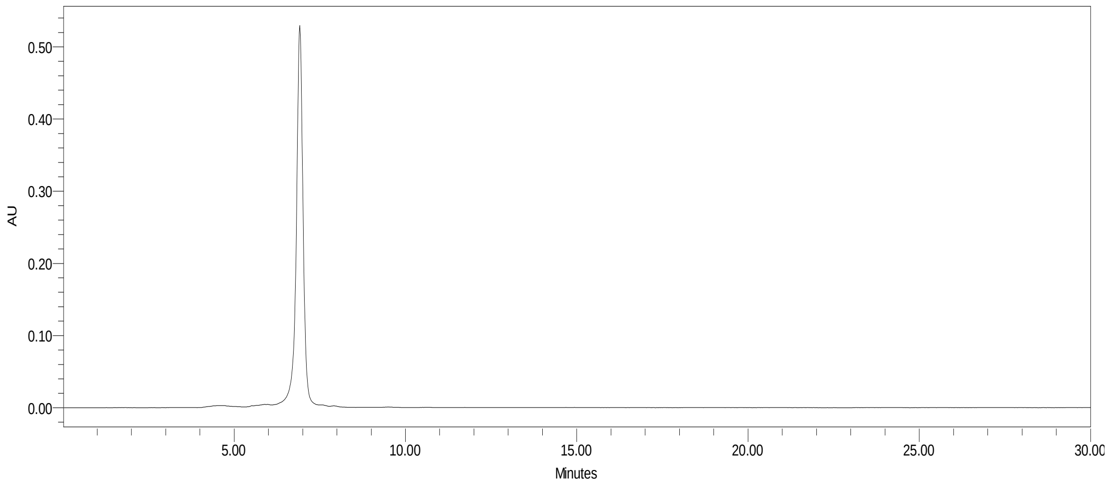

HPLC spectra of A7

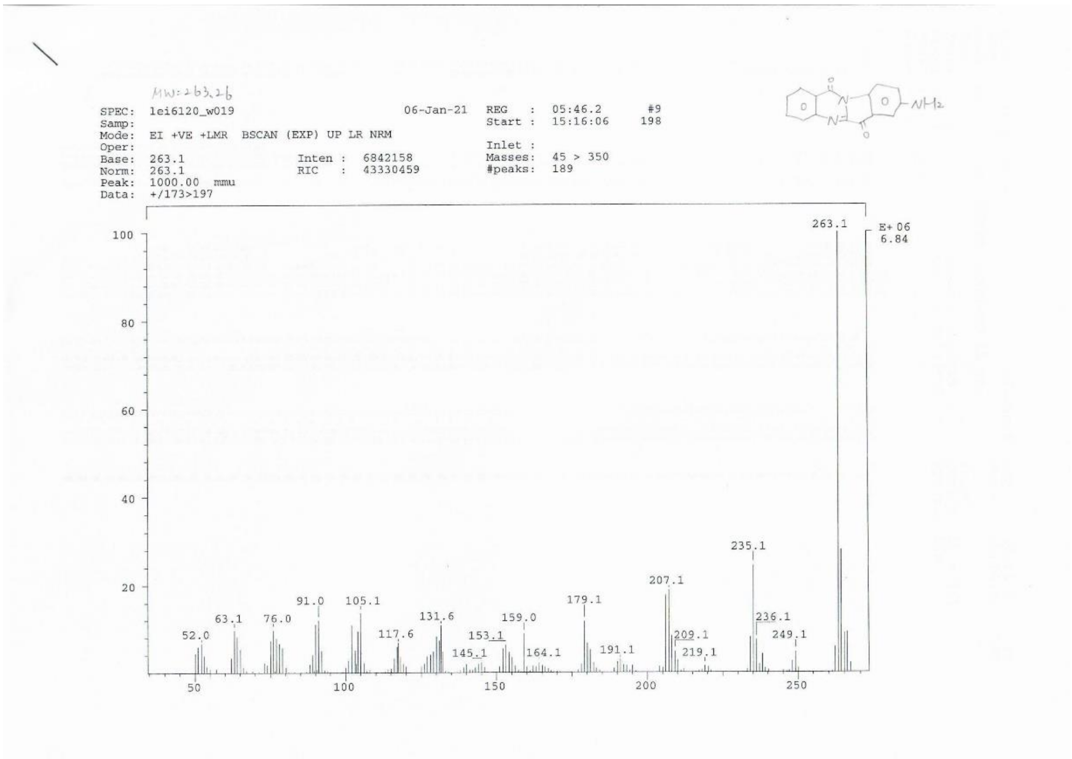

Mass spectra of A7

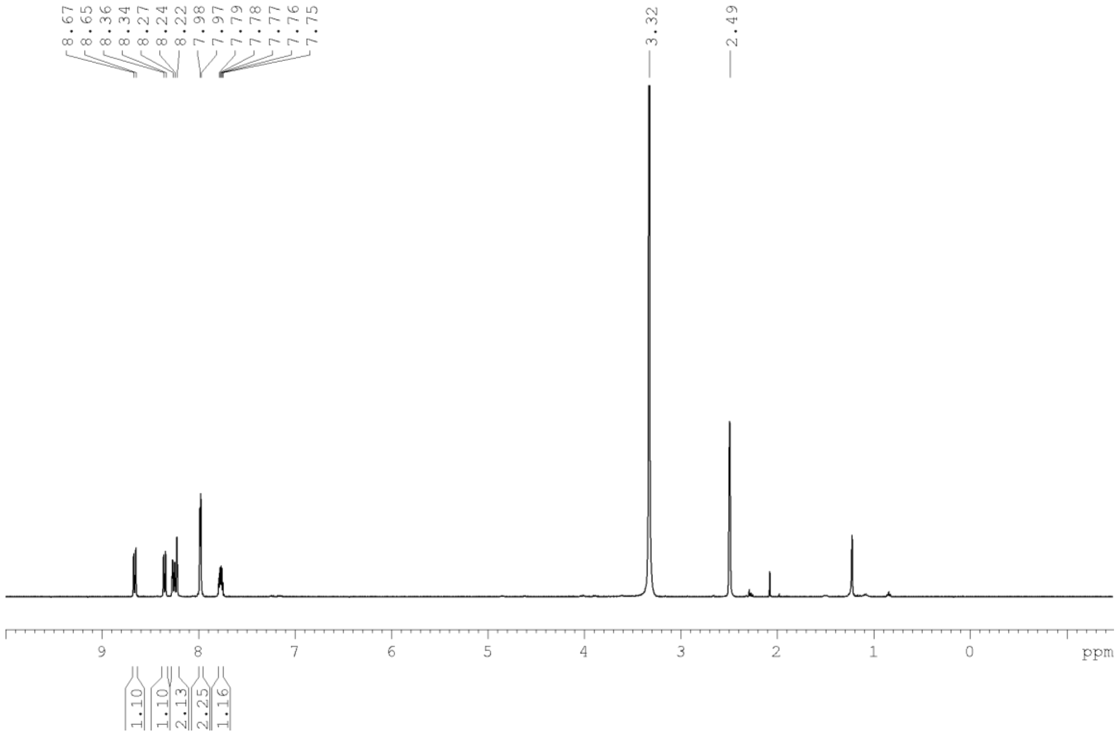

^{1}H NMR spectra of A8

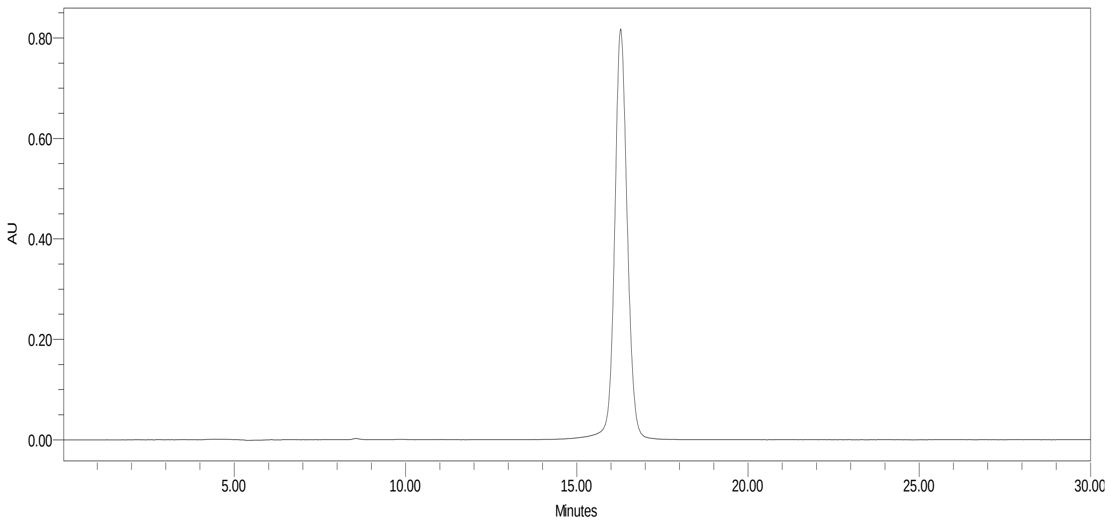

HPLC spectra of A8

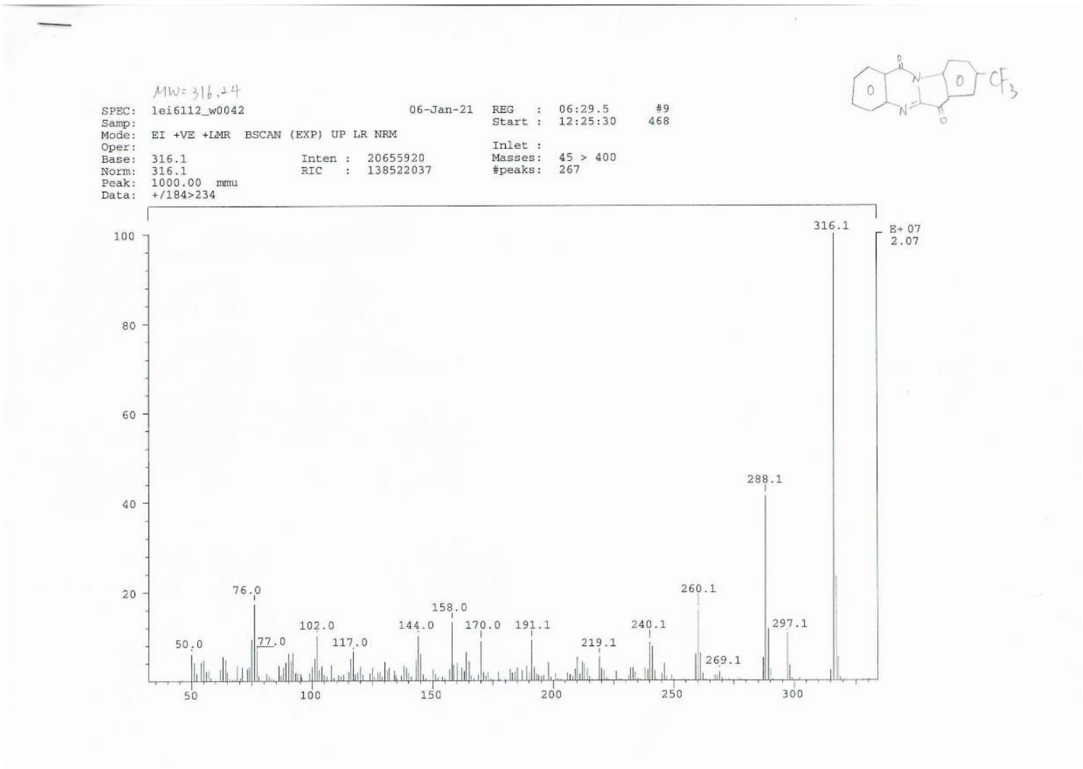

Mass spectra of A8

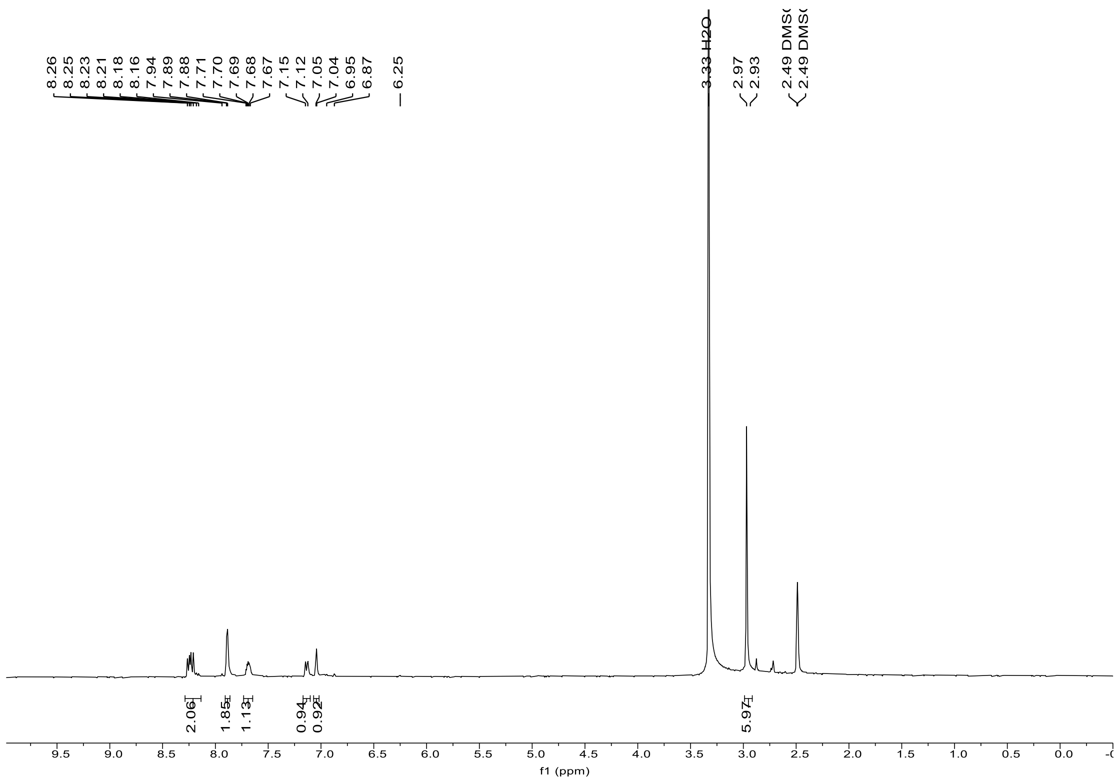

^{1}H NMR spectra of A9

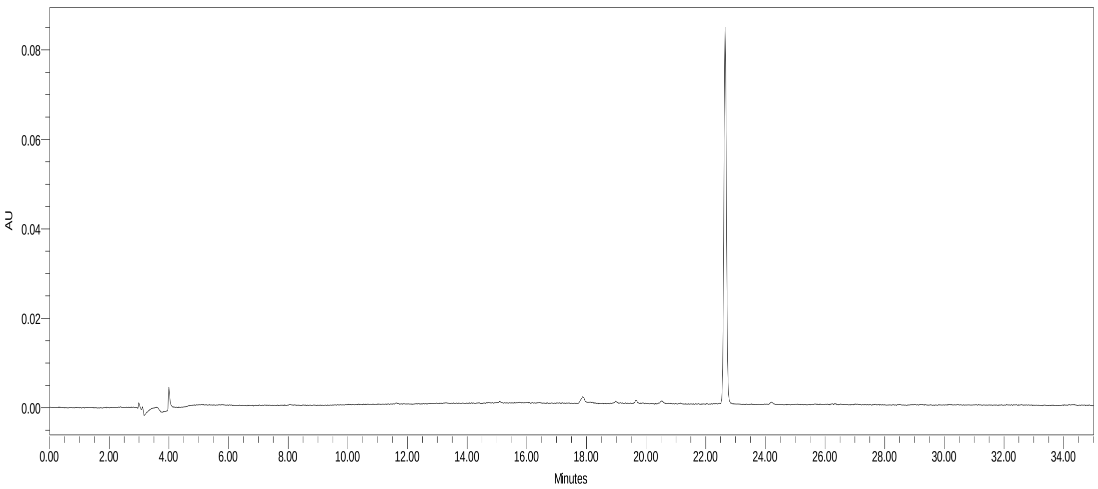

HPLC spectra of A9

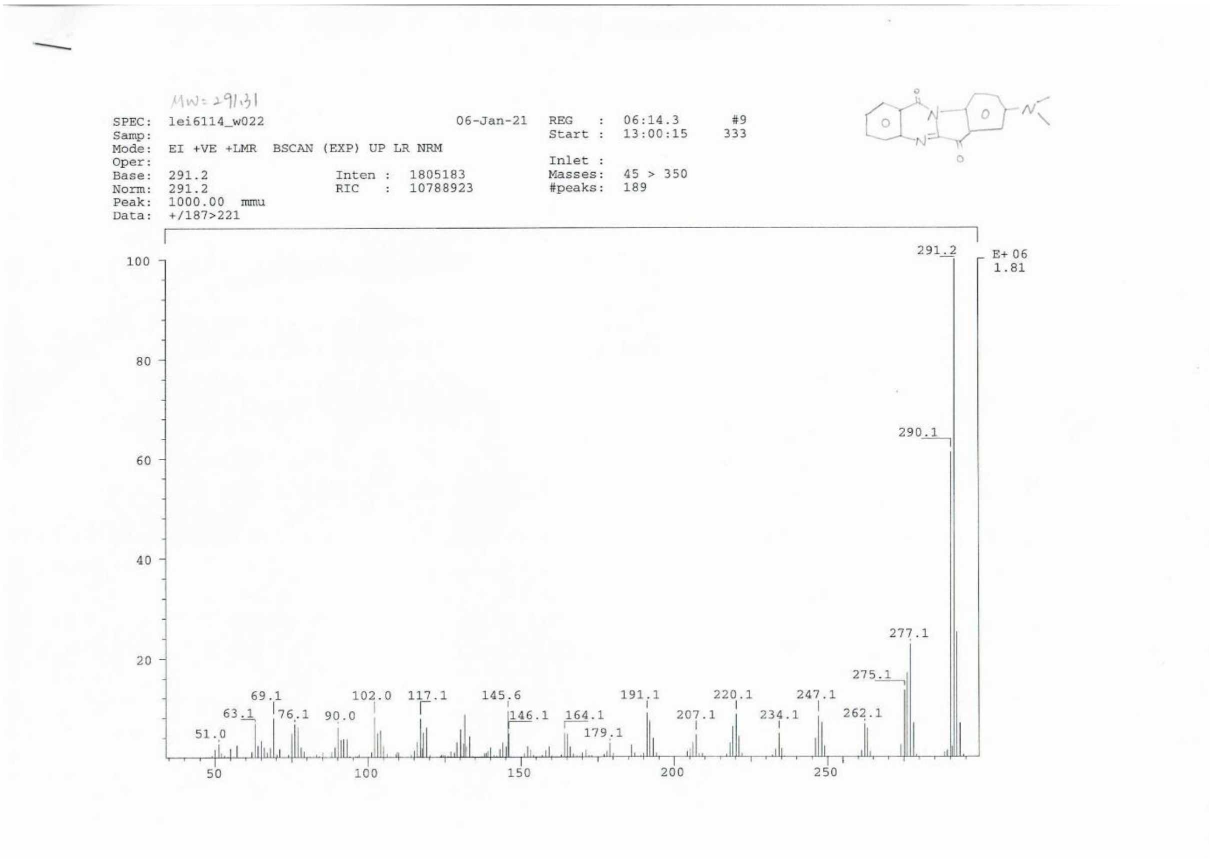

Mass spectra of A9

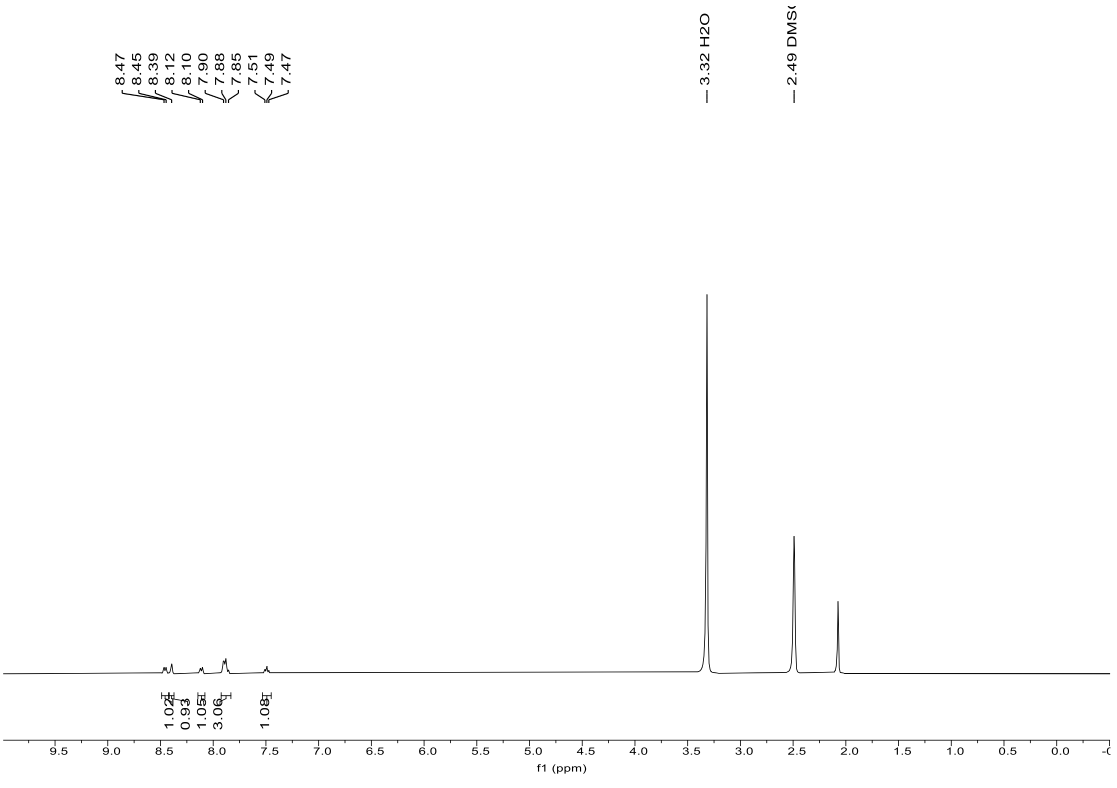

^{1}H NMR spectra of A10

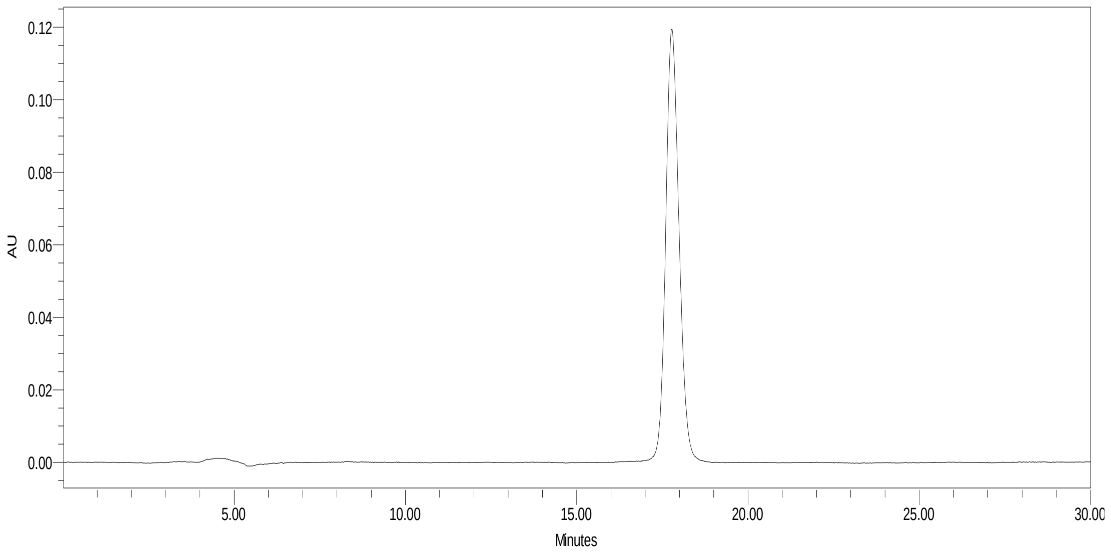

HPLC spectra of A10

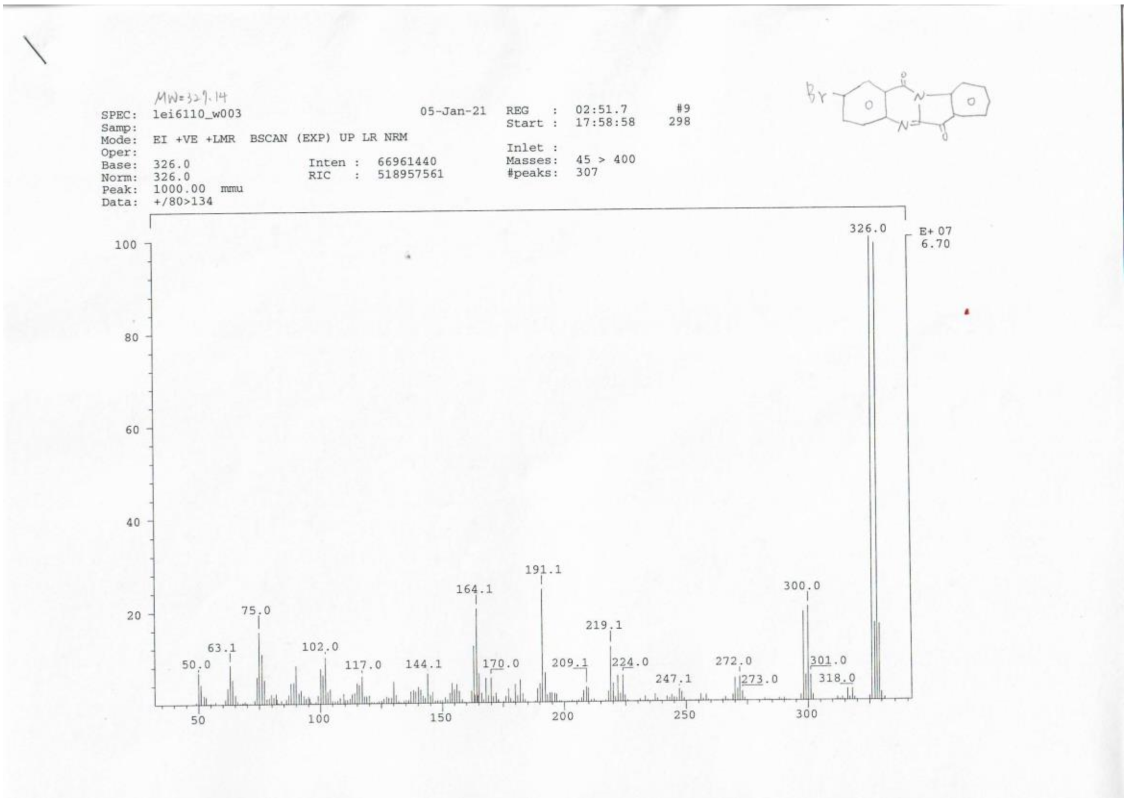

Mass spectra of A10

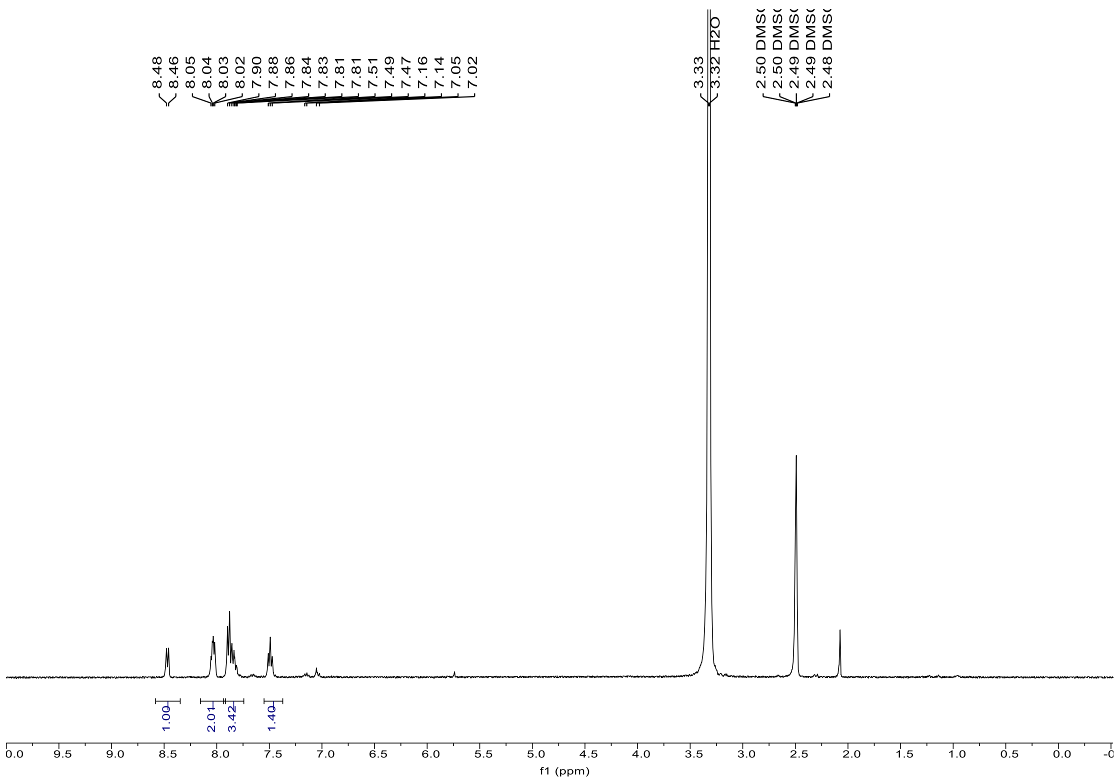

^{1}H NMR spectra of A11

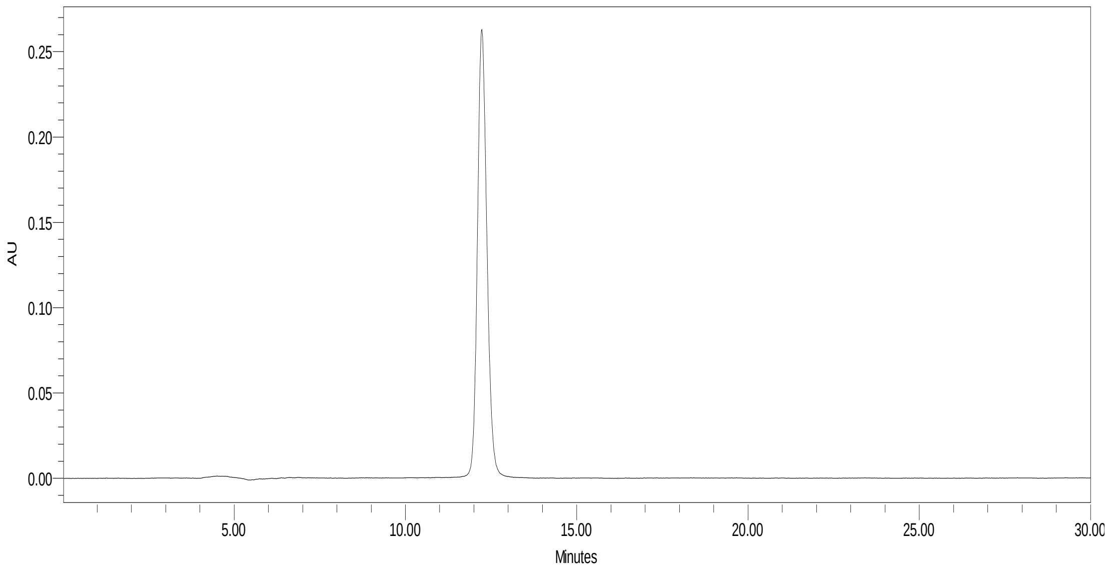

HPLC spectra of A11

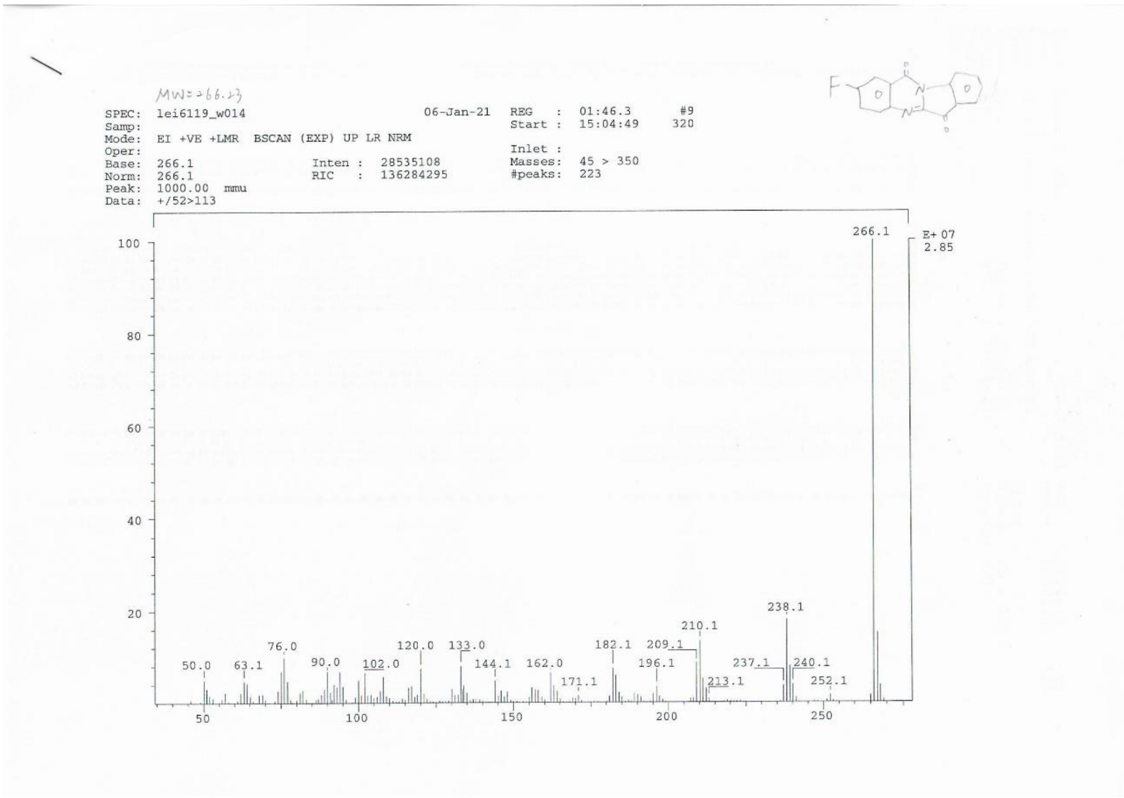

Mass spectra of A11

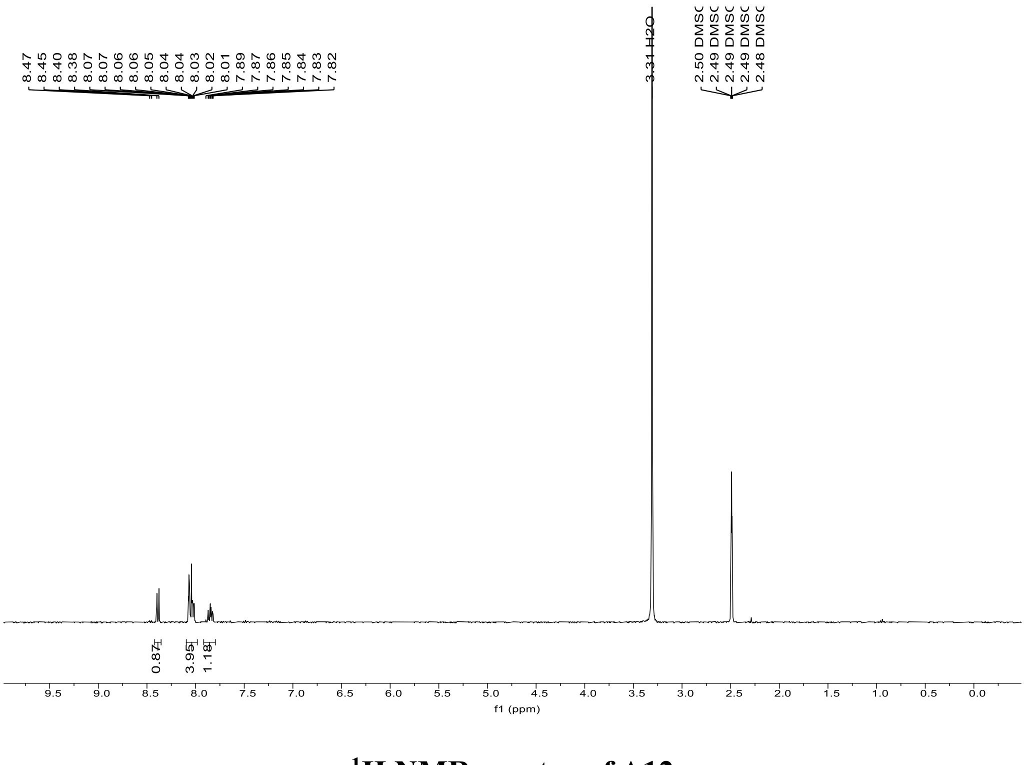

^{1}H NMR spectra of A12

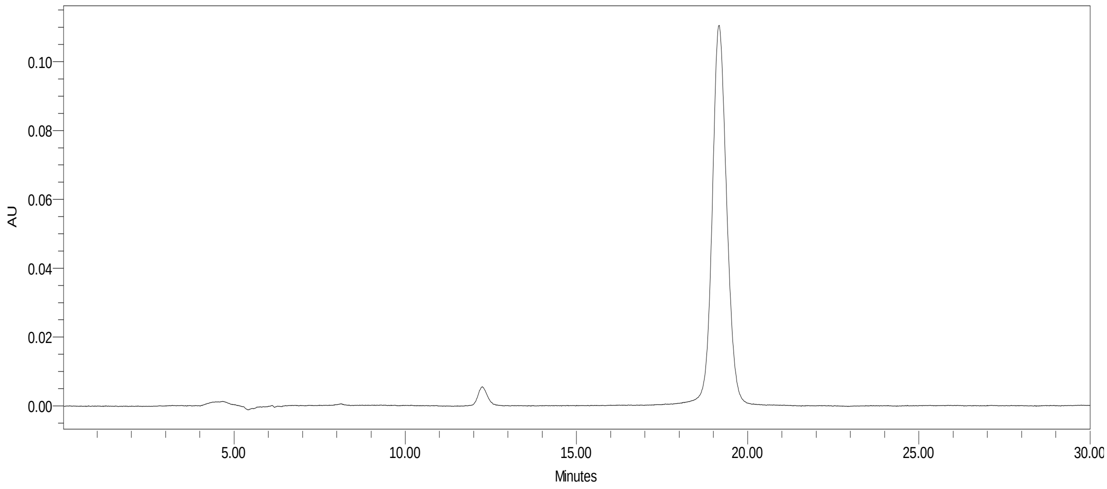

HPLC spectra of A12

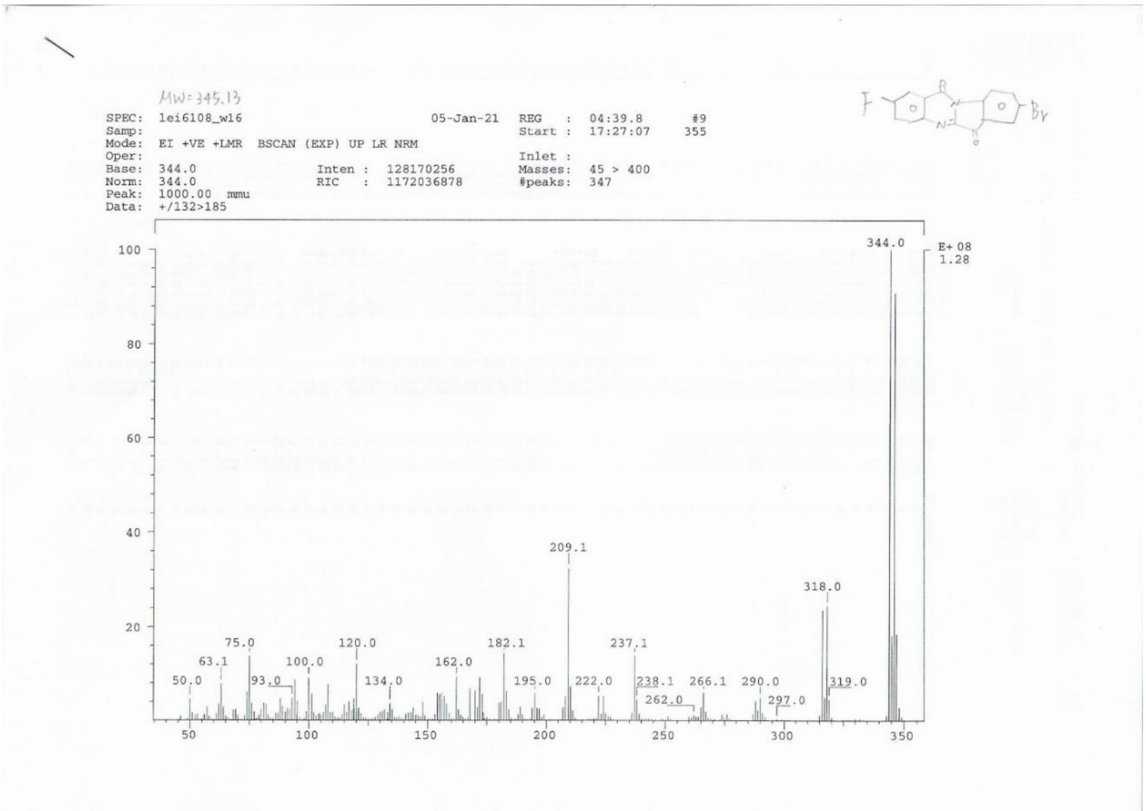

Mass spectra of A12
